# An agent-based approach for modeling dynamics of contagious disease spread

**DOI:** 10.1186/1476-072X-8-50

**Published:** 2009-08-05

**Authors:** Liliana Perez, Suzana Dragicevic

**Affiliations:** 1 Spatial Analysis and Modeling Laboratory, Department of Geography, Simon Fraser University, 8888 University Drive, Burnaby, BC, V5A 1S6, Canada

## Abstract

**Background:**

The propagation of communicable diseases through a population is an inherent spatial and temporal process of great importance for modern society. For this reason a spatially explicit epidemiologic model of infectious disease is proposed for a greater understanding of the disease's spatial diffusion through a network of human contacts.

**Objective:**

The objective of this study is to develop an agent-based modelling approach the integrates geographic information systems (GIS) to simulate the spread of a communicable disease in an urban environment, as a result of individuals' interactions in a geospatial context.

**Methods:**

The methodology for simulating spatiotemporal dynamics of communicable disease propagation is presented and the model is implemented using measles outbreak in an urban environment as a case study. Individuals in a closed population are explicitly represented by agents associated to places where they interact with other agents. They are endowed with mobility, through a transportation network allowing them to move between places within the urban environment, in order to represent the spatial heterogeneity and the complexity involved in infectious diseases diffusion. The model is implemented on georeferenced land use dataset from Metro Vancouver and makes use of census data sets from Statistics Canada for the municipality of Burnaby, BC, Canada study site.

**Results:**

The results provide insights into the application of the model to calculate ratios of susceptible/infected in specific time frames and urban environments, due to its ability to depict the disease progression based on individuals' interactions. It is demonstrated that the dynamic spatial interactions within the population lead to high numbers of exposed individuals who perform stationary activities in areas after they have finished commuting. As a result, the sick individuals are concentrated in geographical locations like schools and universities.

**Conclusion:**

The GIS-agent based model designed for this study can be easily customized to study the disease spread dynamics of any other communicable disease by simply adjusting the modeled disease timeline and/or the infection model and modifying the transmission process. This type of simulations can help to improve comprehension of disease spread dynamics and to take better steps towards the prevention and control of an epidemic outbreak.

## Background

Spatial epidemiology issues are outstandingly important, particularly the viral spread through populated areas is believed to be one of the major concerns [1]. The incidence and prevalence of infectious diseases in a given population, with varied geographic and demographic settings, need to be analyzed over the spatial and temporal domain in order to build dynamic models that provide a global insight of outbreaks' behaviour.

Transmission of an infectious disease may occur through several pathways: by means of contact with infected individuals, by water, airborne inhalation, or through vector-borne spread. However, for the purpose of this study, the direct contact of susceptible individuals with an infected one will be considered as the main transmission medium of contagious diseases. Therefore, it is assumed that infectious diseases are diffused from individual to individual following a network of contact between them. Since this contact usually takes place in a geographical space, it is fairly natural to expect that the space plays an important role in the dynamics of infectious diseases [2]. Clear evidences that some infectious diseases in humans populations spread geographically are the three well-known recent examples of communicable disease spatial advance in the United Kingdom [3] and Canada [4,5]. For this reason, it is required to understand the complex dynamics of contagious illnesses given certain spatial environments. Some of the most well known mathematical approaches are the differential equation models (DE) [6], and mean-field type models (MF) [7], which have not taken into account spatial and temporal factors such as variable population density and dynamics, and they also ignore space implications within the system. The neglect of the spatial component in the formulation of epidemic models can be solved by describing the spatial behaviour with the use of complex systems theory approaches.

One of the challenges that face geographers, epidemiologists and computer scientists working in the field of spatio-temporal modeling, is trying to understand the complexity of the spread of diseases. The search for an understanding of the non-linear behaviour of epidemics' spread and their causes in order to control them, has resulted in several attempts to model and predict the pattern of many different communicable diseases through a population. Models can be defined as an abstraction of the real world, regardless of type or complexity, they are basically simplifications of a real-life system, which can contain only some of the essential elements of it – as determined by the researcher -, models are not exact reproductions of reality and can be interpreted by different people in different ways [8]. In spatial epidemiology, models have been primarily used to facilitate an understanding of the complexity of the interaction between the spread of a disease among different individuals and its impact on society.

It is for these reasons that the objective of this study is to develop and implement an agent-based modeling approach for the spread of a communicable disease. The theoretical framework will be implemented in a case study of measles to allow the creation, representation and execution of a communicable disease propagation simulation over space and time and in an urban environment. One of the most important factors that this study considered is the complexity of mobile individuals in an urban setting with transportation network, their exchanges during the commuting time and some of the possible interactions among them in specific locations such as work places, schools, university and shopping malls where people flow and where their contacts and interactions are dynamic.

### Representation of Space and Time in Epidemic Modeling

Epidemics have been modeled making use of many different types of models, from those that are purely mathematical to the spatially explicit ones. The mathematical modeling of epidemics has been the subject of a number of studies over the past century [9]. The formulation of these classic epidemic models enable the simulation of events for which laboratory experiments could not be conducted easily. The main assumption of this kind of models is that the population, in which a pathogenic agent is active, comprises different subgroups of individuals and they examine only the temporal dynamics of the infection cycle.

### Classic Epidemic Spread Models

Traditional epidemiology models represent epidemics of communicable disease using a population-based, non-spatial approach. The conceptual framework for this approach is rooted in the general population model which divides a population into different population segments [10]. Nowadays, epidemiology has known numerous disease-spreading models; one of the most famous models is the stochastic model introduced by Kermack and McKendrick (1927) [11], followed by others more or less sophisticated. The simplest model of epidemic spread which employs deterministic ordinary differential equations, is based on the separation of the total population into two groups: "Susceptible" (those individuals who are potentially capable of contracting the disease), and "Infected" (those individuals who are capable of spreading the disease). Due to this division of the population the model is called "SI". There are other epidemic models also based on the classification of the total population (SIR: Susceptible-Infected-Recovered, SEI: Susceptible-Exposed-Recovered, and SEIR: Susceptible-Exposed-Infected-Recovered). These deterministic models assume that populations are completely mixed and ignore spatial effects of spread epidemics; also interaction between individuals is neglected since they model populations as continuous entities [12]. The SI, SIR, SEIR, SIS and SIRS models fail to effectively model spatial aspects of the spread of an epidemic, the individual contact process, and the effects of individual behaviours, among others [12]. For this reason, the development of new mathematical and computing methodologies are necessary.

### Complex Systems Approaches for Epidemic Spread Models

Cellular automata (CA) theory has been used for modeling location-specific characteristics of susceptible populations together with stochastic parameters that capture the probabilistic nature of disease transmission [13,14]. However, the representation of individuals' movement and interactions over the space was no presented. This is an important factor to consider in highly contagious diseases and therefore this methodology gave way to a new approach. Agent-based modeling (ABM), is also a bottom-up approach, similar to CA models, but has the advanced capability of tracking the movement of a disease and the contacts between each individual in a social group located in a geographic area [15,16]. The potentials that ABM possess to model epidemic spread, have been used in epidemiology to study and track the movement of infected individuals and their contacts in a social system [17,18].

Agent-based models allow interaction among individuals and are capable to overcome the limitations of different approaches such as cellular automata and classical epidemic models, permitting to study specific spatial aspects of the spread of epidemics and addressing naturally stochastic nature of the epidemic process. Consisting of a population of individual actors or "agents", an environment, and a set of rules [19], actions in ABM take place through the agents, which are simple, self-contained programs that collect information from their surroundings and use it to determine how to act [20]. Modeling in epidemiology using an agent-based approach pursues the progression of a disease through each individual (thus populations become highly heterogeneous by health status during simulations), and tracks the contacts of each individual with others in the relevant social networks and geographical areas (e.g., co-workers, schoolmates). All the rules for individual agent movement (e.g., to and from workplace and/or school) and for contacts with and transmissions to other people are explicit [21].

ABMs and their ability to produce emergent macro-effects from micro-rules have served as a cornerstone for the development of different methodological frameworks in epidemiology [16]. Epidemiologic applications using ABM approach are mostly designed to allow epidemiological researchers to do a preliminary "what-if" analysis with the purpose of assessing systems' behaviour under various conditions and evaluating which alternative control policies to adopt in order to fight epidemics such as smallpox [22-24]. Although these models effectively track the progression of the disease through each individual, and track the contacts of each individual with others in the relevant system (social or natural), they need to add physical infrastructures such as road networks, and real geographic environments to account for more complex interactions among susceptible and infected individuals. Another important application of ABM in epidemiology is the modeling of vector-borne diseases and the changes in their incidence that are attributable to climatic changes. These models have been developed in order to allow the evaluation of impacts of climate change on vector borne diseases like malaria, as well as the a priori evaluation of environmental management-based interventions [25].

The spread of human epidemics strongly relies on the structure of the underlying social network, and it has become clear that differently structured networks lead to different types of epidemiology [26,27]. By modeling the correlations between individuals, it is possible to understand the role of spatial heterogeneity in spreading dynamics. The previous statements have lead to the development of different models in order to depict the spatial behaviour of diverse infectious diseases through structured and realistic urban networks, for example, influenza [10,18,28-31], Mumps [32,33], West Nile virus [34,35], Tuberculosis (TB) [16], Lassa virus [18], among others. Some of the models mentioned represent the spatial distribution and mobility of individuals making it possible to model the spatial heterogeneity in the disease transmission. Nonetheless, one of their drawbacks is the lack of use of real landscape structures and integration with geospatial data and geographic information systems (GIS) to represent the continuous environment where the discrete individuals interact.

## Methods

Communicable diseases are illnesses caused by an infectious agent that are highly contagious and may be transmitted from one individual to another one through direct contact. Individuals that make part of a human population are involved in a sequence of activities on a daily basis. Some of the activities are stationary and some are mobile. Stationary activities occur at fixed locations, such as a home, school, workplaces, commercial and shopping areas. At these geographical locations, individuals may interact among themselves in a group activity. Mobile activities are related to the daily commuting activities of individuals through the public transportation system. When a group disperses, an individual travels through space and time to a different location, often interacting and joining another group. The simulation of this population dynamic is essential to depict individuals' life path of movement through space and time. The disease propagation modeled in this study represents this movement path as a trajectory in space (movement from one place to another) through a transportation network and in time, expressed on hourly basis. In this fashion the daily activities of commuting, studying, working and leisure time are simulated.

The methodology for this study involved the development of a complex algorithm composed by two parts. The first was designed to describe communicable disease stages, which is the generic infection model. The second represents the rules that govern the life path behaviour of the agents and the infection behaviours that allow the transmission of the disease within a group of people in a city. Two scales are considered for the individual interactions in respect to transmission and propagation of the disease. One is at the individual scale considering the smallest space around a person when the disease can be transmitted. Second is the limit of boundaries of the city in which individuals move, live and interact with each other on a daily routine.

### Generic Infection Model

**Figure 1:**
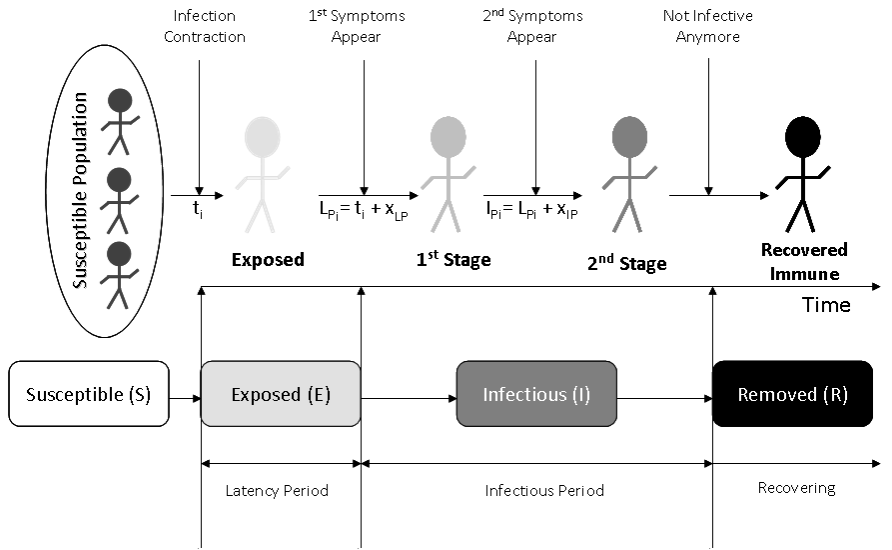
**Different states of the SEIR infection model, to simulate the progress of and epidemic in a human population**. L_Pi_: latency period, I_Pi_: infectious period, t_i_: first day that an individual is exposed to the virus for the first time, x_LP_: number of days for an exposed individual to become infective, and x_IP_: number of days for an individual to recover from the disease.

Highly communicable diseases may spread by airborne droplets or through direct contact with nasal or throat secretions emitted through sneezing or coughing of infected persons [36]. Symptoms may vary between diseases, but they generally are divided in two stages. In the first stage, after the exposure to the infectious agent the symptoms are nothing special and used to be associated with a typical cold. The second stage initiates after a certain number of days and it is at that time when the specificity of the disease is very evident. After examining different compartmental models used in epidemiology, this simulation adopted the Susceptible-Exposed-Infectious-Removed (SEIR). The SEIR model successfully addresses a significant period of time during which an individual has been exposed to the infection but is not yet contagious: *Exposed *phase or latency period. The latency period is used to describe the period of time between exposure to the virus and the time the disease becomes apparent through symptoms and signs. The morbid period, also known as *Infectious *phase, is the period of time between the moment an individual starts being infectious until it is recovered (Figure 1).

In this study, for simplicity reasons, the model is developed in such way that all recovered individuals cannot become infected again and they will remain immune. The initial conditions for the simulation are set up to represent a susceptible population, an immune population (due to previous communicable disease infection or vaccination), and infected population at the beginning of the outbreak. In order to design and implement the generic model, mathematical expressions are developed, so that the model can be used for different types of communicable diseases. As depicted in figure 1, latency period (L_Pi_) and infectious period (I_Pi_) are presented with following equations:

(1)

(2)

where (**t**_**i**_) represents the one single day that an individual is exposed to the virus for the first time; (**x**_**LP**_) represents the number of days that have to elapse before the exposed individual to become infected; (**x**_**IP**_) corresponds to the number of days it takes for the individual to recover from the disease; (**x**_**LP**_) and (**x**_**IP**_) take different values for different diseases.

**Figure 2:**
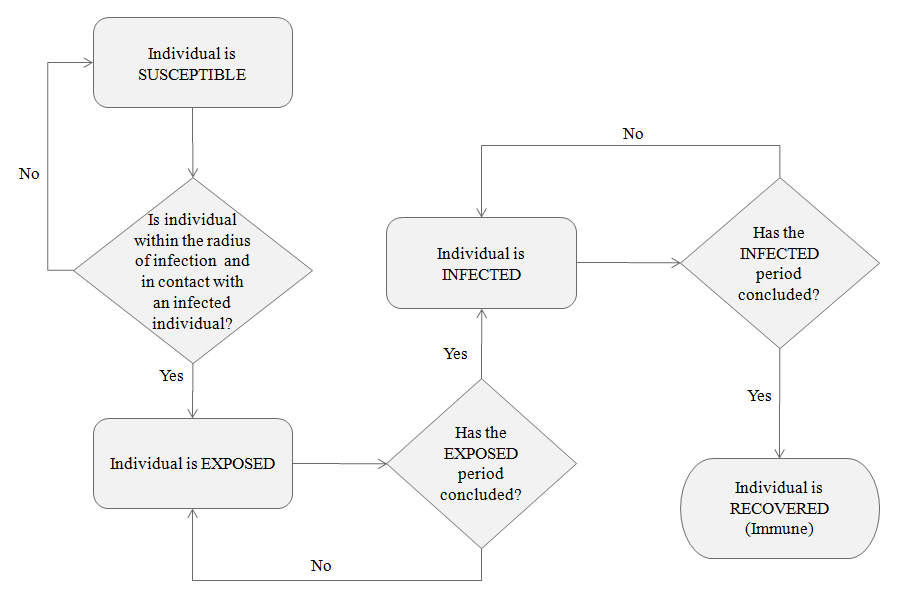
**Flow diagram representing different infection phases**.

The rules that govern the unconscious biological process of disease spread are depicted in the flow diagram presented in Figure 2, where each individual is represented as an agent in the proposed model. Once an infectious individual arrives at a fixed location to perform any stationary activity (e.g., study, work, shopping, etc) a calculation is performed to determine the number of susceptible individuals within its perimeter to be contagious (Pc). This area is calculated based on a radius (*r*) of 1 m distance from the infected individual, considered as the smallest distance at which an individual can contract the disease and get infected.

(3)

After determining the susceptible individuals within the infected persons' surroundings (Pc), the disease is transmitted to some of them.

### Agent Based Model

**Figure 3:**
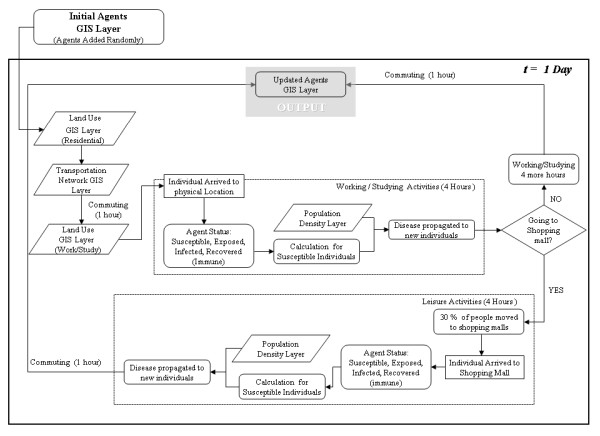
**Process of the epidemic spread AB model for a single time step representing daily activity of individuals' activities and their interactions in an urban environment**.

The model proposed in this study attempts to realistically represent the behaviour of individuals' daily path in an urban setting, as well as characterize the natural biological process of the disease spread among individuals (Figure 3). So that, it is required to maximize the simplicity regarding the interactions that one can account between agents, which then allow maximizing the understanding of their dynamics. The agent based model operates on discrete time steps during which a population of individuals, represented as agents, moves through a geographical space, where daily activities are performed. In order to represent a normal daily routine, it is assumed that the length time for out-of-house daily activities for an individual is 10 hours. Two hours are spent commuting via public transportation, and the other eight hours are spent either in work places, study places (high schools, universities, community colleges, etc) or doing some leisure activities at places like shopping malls.

**Figure 4:**
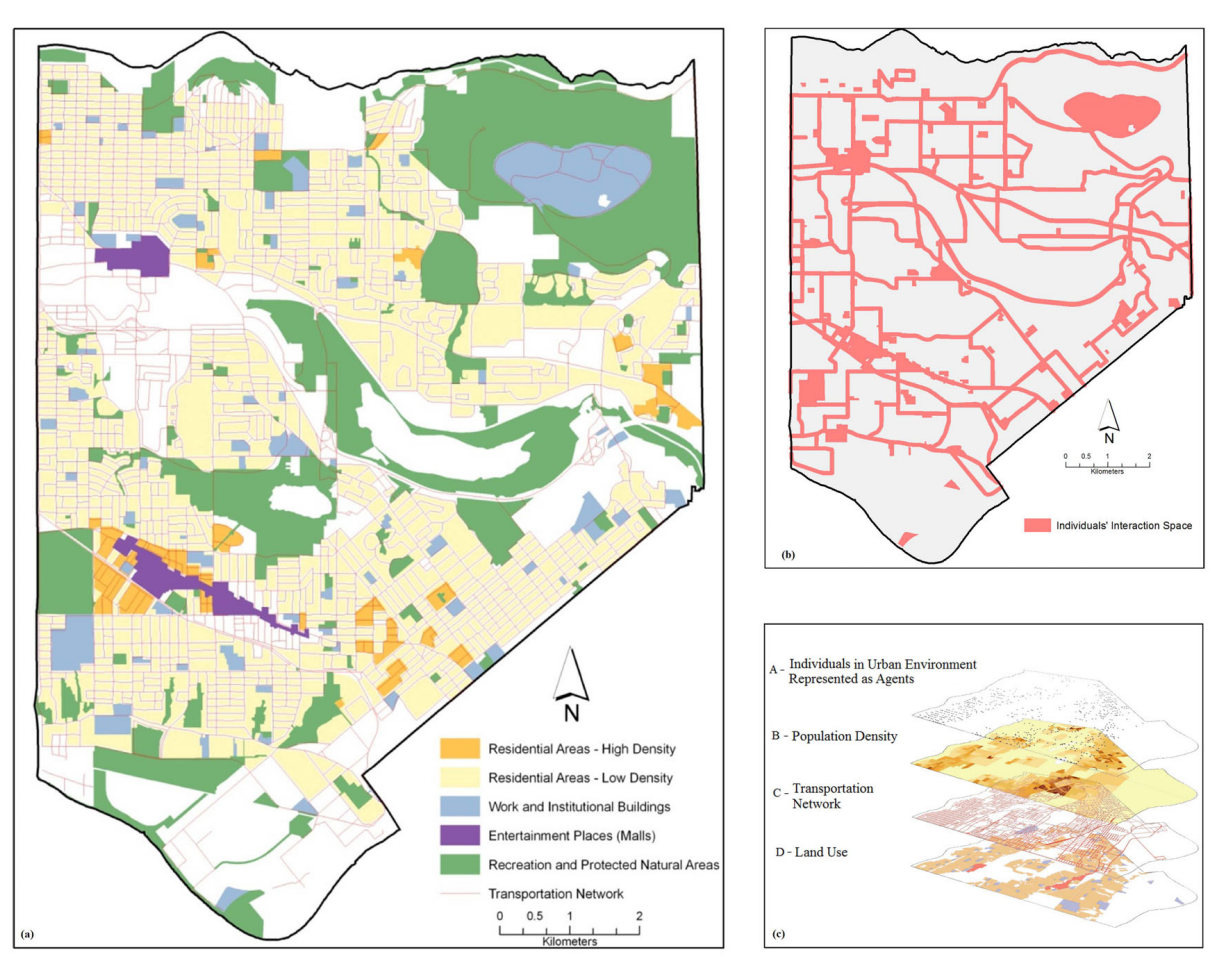
**(a) Geographic area: City of Burnaby, Canada, with the relevant land use classes; (b) Geographic space of individual's interactions; (c) Geospatial data inputs**.

ABMs are best described in order of their major components: environment, agents and interactions. This model is implemented using georeferenced GIS data layers of an urban area in order to geographically represent the usual urban landscape where typical individuals' contact takes place. In order to account for some of the factors that may influence an epidemic in urban areas, the model was designed to include georeferenced information of population densities, different land uses and transportation networks. The flow diagram designed to represent the process of the disease propagation is used to explain the method for searching and querying the different geographic layers to determine the behaviour of the agents. As shown in figure 4(c), the agent-based model has four different georeferenced inputs; the first one (A) is the population of agents that represent the behaviour of individuals living in an urban area. The second (B) is the information regarding population density; this dataset allows the evaluation of the number of people per square meter and thereby greater probabilities of getting the infection will be assigned to individuals that perform any stationary activity in these areas. The third (C) is the transportation network, which is used by the agents to commute from one place to another within the municipality. Finally the fourth input (D) represents three different land use types: residential areas (houses, townhouses and apartments), work and study areas (institutional and commercial buildings), and entertainment areas (shopping malls).

**Figure 5:**
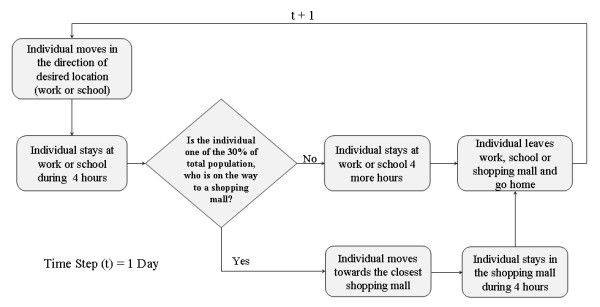
**Flow diagram that characterizes daily activities of individuals within the city**.

Only one type of agent is used in this model to represent individuals and it has no attributes of age or gender. When the model is initialized and the agents are randomly added to residential areas, the georeferenced location of each individual is established and stored in the individuals' memory to recall its point of origin. After this initial stage, the agents simulate individuals' daily commuting to work places or study buildings. The entire susceptible population is divided into 70% of workers and 30% of students. After the first commuting hour, the workers go to work places and the students go to study areas. Figure 4(b) depicts the closed geographic space where individuals interact amongst themselves and transmit the disease. The movement of the individuals through the transportation network simulates the commuting behaviour, and was accomplished using a geographic information system (GIS) that holds the spatial locations of all the roads and stores the topological relationships between them. Therefore, if an agent needs to get to a destination, it needs to search the transportation network dataset in order to know which roads it has to travel along and then actually plot the shortest route to get to the desired point. The routing algorithm works by firstly building a list of coordinates which the agent must pass through to get to its destination and then moving along the planned route. The flow diagram shown in figure 5 illustrates the behaviour that determines different activities performed by diverse individuals.

The agents are mainly distinguished by their health status (e.g. susceptible, exposed, infected, immune), movement rules, and mode of infection transmission. For the purpose of this model simulation, only public places (work places, schools and shopping malls) were considered for the propagation of the disease, this implies that the infection rules are not effective during the night time period in the residential areas or elsewhere.

**Figure 6:**
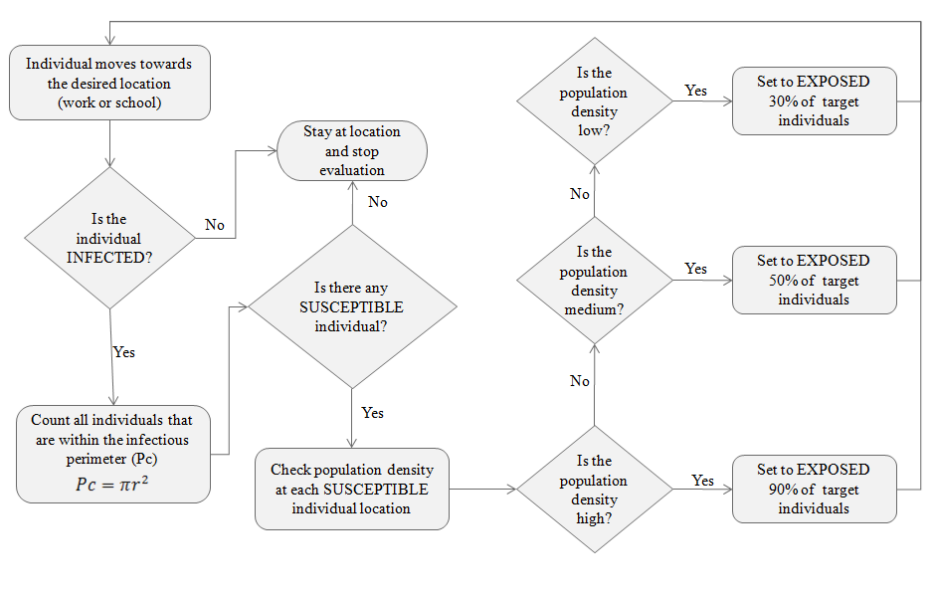
**Flow diagram for the infection rules that describe the disease propagation among individuals at physically fixed location**.

An infection can only be transmitted from an infected person in a contagious stage to individuals that are healthy (susceptible) and within the established perimeter (Pc) for the disease to be communicable – explained in equation 3. The total number of individuals that are exposed within this perimeter (Pc) depends also on the population density where they are located at the moment of the interaction with and infected agent. If a group of susceptible individuals interact within the perimeter (Pc) of an infected individual, not all of them will contract the disease and therefore the rate of infection is determined using low, medium and high population densities, in order to calculate the number of individuals that are exposed to the disease. The fact that disease spread risk is considered to be higher for individuals living in highly dense populated urban areas is therefore taken in consideration [37]. Assuming a direct relationship between population and rate of infection, higher rates are given to those individuals located in areas with high density population. The flow diagram which describes the infection model and therefore the disease propagation among individuals is presented in Figure 6.

The population of agents is held constant during a simulation run. Even if they have recovered, immune agents are not removed from the population. Agents are thus characterized by their location in the environment and by their internal state (status), which can be: susceptible, exposed, infected or recovered (immune). Once an agent is exposed, it remains infected for certain amount of days until it loses the infection status and recovers. This individual, therefore, remains immune for the rest of the simulation.

## Model implementation

### Case Study

For implementation purposes a simulation of measles epidemic in a human population located within the city of Burnaby, BC, Canada, is used to implement and illustrate the methodological framework. There is clear evidence that infectious diseases in human populations spread geographically. A known example of such communicable disease spread is the measles outbreak in the metropolitan area of Metro Vancouver in British Columbia, Canada. On January 28th 1997, three cases of measles among students attending a public university in the city of Burnaby were initially reported to the British Columbia Centre for Disease Control; by April 1st 1997, 107 cases of measles had been confirmed to be spread in some surrounding areas linked to Simon Fraser University (SFU) [38]. Measles (also known as rubeola) is a disease caused by a virus, specifically a paramyxovirus of the genus Morbillivirus. This infectious disease spread through contact with fluids from an infected person's nose and mouth, either directly or through aerosol transmission. Measles is highly contagious, and it is known and stated by epidemiologists that 90% of people without immunity sharing daily activities with an infected person will catch it [36].

### Geospatial Data Sets

Simulation of a measles epidemic spread in a geographic area is computationally intensive and requires the use of georeferenced data sets and a limited number of individuals interacting in the urban space. For computational simplicity, the use of geospatial data for the City of Burnaby, Canada has been chosen to implement the model. The selection of the area was made based on the structure of the area and its dynamics. Burnaby is the city immediately east of Vancouver and makes part of the Metro Vancouver district. It is the third-largest and most populated urban center in British Columbia. The city features high and low density residential areas, major commercial town centers, industrial complex, rapid transit, and major post-secondary institutions including one public university and a technical institute (Figure 4). The complexity of urban dynamics within the City of Burnaby makes is a suitable study site to implement the proposed model. Geoferenced data sets are derived from 2001 population census data from Statistics Canada [39] for population densities, from Metro Vancouver [40] for land use data and from the Greater Vancouver Transportation Authority (Translink) [41] for transportation network data.

### Agent-based Simulation Toolkit

In order to implement the designed ABM, Repast Simphony (RepastS) and some of its Java libraries [42] were used. RepastS extends the Repast portfolio by offering a new approach to simulation development and execution, including a set of advanced computing technologies for applications such as disease spread simulation. In addition to the integrated library of classes for agent-based simulations, this toolkit allows simulations within a geographic information system environment.

**Figure 7:**
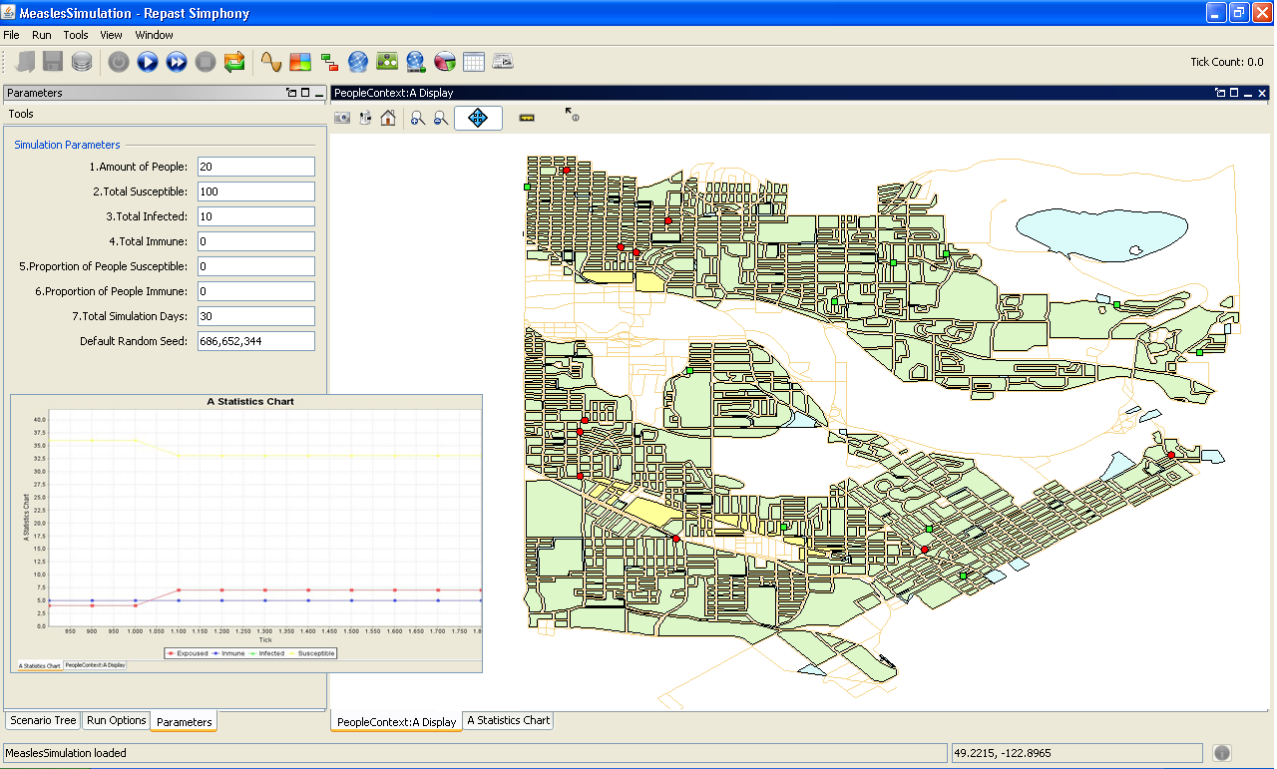
**Graphical user interface (GUI) developed for the model implementation. Dots represent the individuals moving within an urban environment**.

RepastS introduced the context and projection concepts. The context is basically a bucket that is used to hold a population of agents but does not give agents any concept of space or relationships. Once the agents are in a context, projections can give the agents a space and can define their relationships. For example, GIS projections give each agent a spatial location and Network projections allow relationships between agents to be defined (e.g. a transportation network). These new concepts allow the model implementation having agents with a spatial location and movement around a geographic environment using the transportation network. In order to link the agent-based model to a GIS it is necessary to code the instructions that allow the ABM to read the GIS files (shapefiles). Hence, the individuals interact among themselves within the geographic space represented in the model (Figure 7). The agent-based simulation created for this study is a collection of agents and a model that is in charge of setting up and managing the execution of these agents' behaviours and the spatial relationship between them and their urban environment. The developed model is responsible for setting up and controlling simulation visualization, data recording and analysis.

### Implementation

The entire population of the city cannot be taken in consideration due to computational reasons. In addition, it is expected that within the city not everyone is always commuting and interacting at the same time. Only a percentage of the population is in labour force or are students, therefore a limited number of individuals has been chosen for the simulations [39]. The model implementation is accomplished by using 1000 individuals involved in a measles epidemic and interacting at a city scale. The measles SEIR model timeline adopted makes use of 12 days for the latency period and 8 days for the infectious period. Four scenarios are designed to illustrate different ratio of susceptible versus infected individuals: a) Scenario 1: 999 susceptible individuals and 1 infectious individual, b) Scenario 2: 990 susceptible individuals and 10 infectious individuals, c) Scenario 3: 950 susceptible individuals and 50 infectious individuals, d) Scenario 4: 800 susceptible individuals and 200 infectious individuals. The model was tested using a time frame of sixty days for Scenario 1 and thirty days for Scenario 2, 3, and 4 to observe and contrast the evolution and spread of the disease trough time in the study area shown in figure 4.

The graphical user interface (GUI) (Figure 7) was developed to add flexibility to the implementation and model output scenarios visualization capability. The GUI allows different users to create and test various scenarios by changing the total population and the ratio of susceptible, infected and immune individuals that take part of the simulation. Likewise, the time frame can be modified and the GIS layer with the agents' attributes can be stored for statistical analysis and the colours of the display can be also modified by the user. The geographic display in the GUI permits the visualization of urban landscape where the individuals move. Through the GUI simulation parameters can be changed at anytime to visualize new scenarios

## Results and discussion

**Figure 8:**
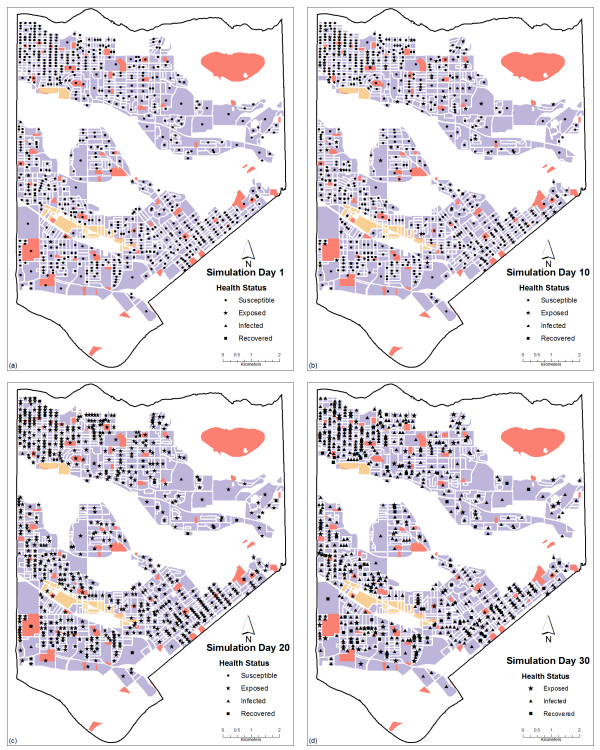
**Spatial distribution of Susceptible-Exposed-Infected-Immune population in an urban area on two different days**. (a) day 1, (b) day 10, (c) 20 and (d) day 30 for Scenario 1. The black circles represent the susceptible population; the black stars represent the exposed population; the black triangles represent the infected population and the black squares represent the recovered (immune) population.

**Figure 9:**
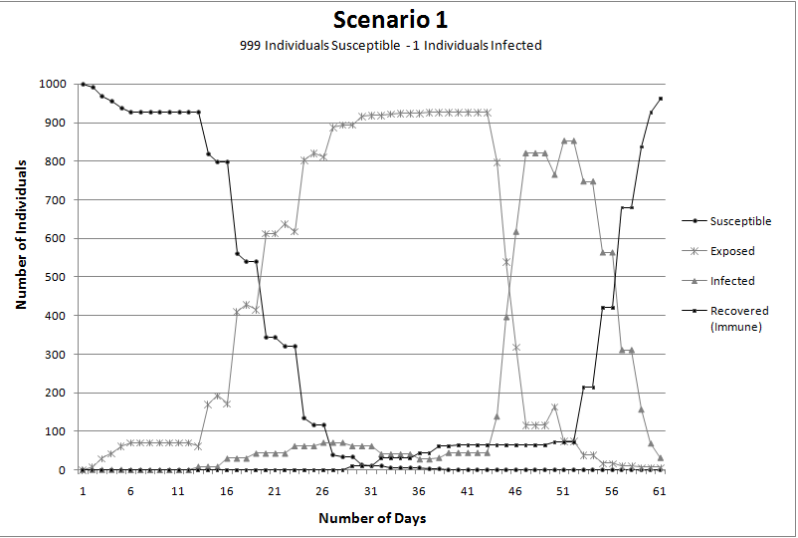
**Graphical representation of the disease spread progression comparison of the proportion of individuals in each health state through time**.

The results are obtained based on simulations for each of the four scenarios. Figure 8 depicts the measles infection progress using Scenario 1, (a) is portraying day one of the simulation, (b), (c) and (d) depict respectively day ten, twenty and thirty of the epidemic spread for a closed population of 1000 people. Figure 9 presents the number of daily exposures, infections and recoveries for a measles epidemic over a period of sixty days for Scenario 1. These results demonstrate that during the first day of interaction amongst population, at least 1% of the population were in contact with the infected individual and therefore was exposed to the virus. Five days after the first contact between some of the population and the infected individual, the rate of contagion remains stable with an average of less than one percent of the population interacting and being exposed to the virus due to the lack of diagnosis and removal of the infected individual from the susceptible population.

From the fifth day to the eleventh day there are no new contagious individuals, but the ones that have been exposed to the virus are about to become infectious to their surrounding coworkers, classmates or people sharing the same free time daily activity (e.g. visiting the mall). The stability in the number of individuals infected between the fifth and the thirteenth day is the result of the stationary activities performed by the individuals who usually share with the same group. This steadiness is also product of low interaction rates within the population through the entire city due to some concentration of exposed individuals in geographical locations like schools and university. Another reason for this steadiness is that the agent-based simulation of the disease propagation depends on the increase of individualized infection life paths to peak or decline over time.

On the thirteenth day the number of individuals exposed decrease from the day before, and this was due to the evolution from the exposed state of health to the infectious state of health. The thirteenth day marked the increment in the infected population and since more individuals were able to transmit the virus, the percentage of the population infected increased from one day to the other by two percent. After day sixteen the number of individuals infected increased almost exponentially. The spatial distribution of the simulated agents located at geographically fixed locations is portrayed in figure 8, representing spatial locations of the individuals during the first, tenth, twentieth and thirtieth day. The agents are initially located at residential areas. After the first hour of the day during which the commuting takes place, agents are relocated to workplaces, schools or university, to interact with their coworkers or classmates that are at the same location. Different health statuses are represented by different shapes to show the stages of the epidemic in a spatial context and also through different moments in time.

**Figure 10:**
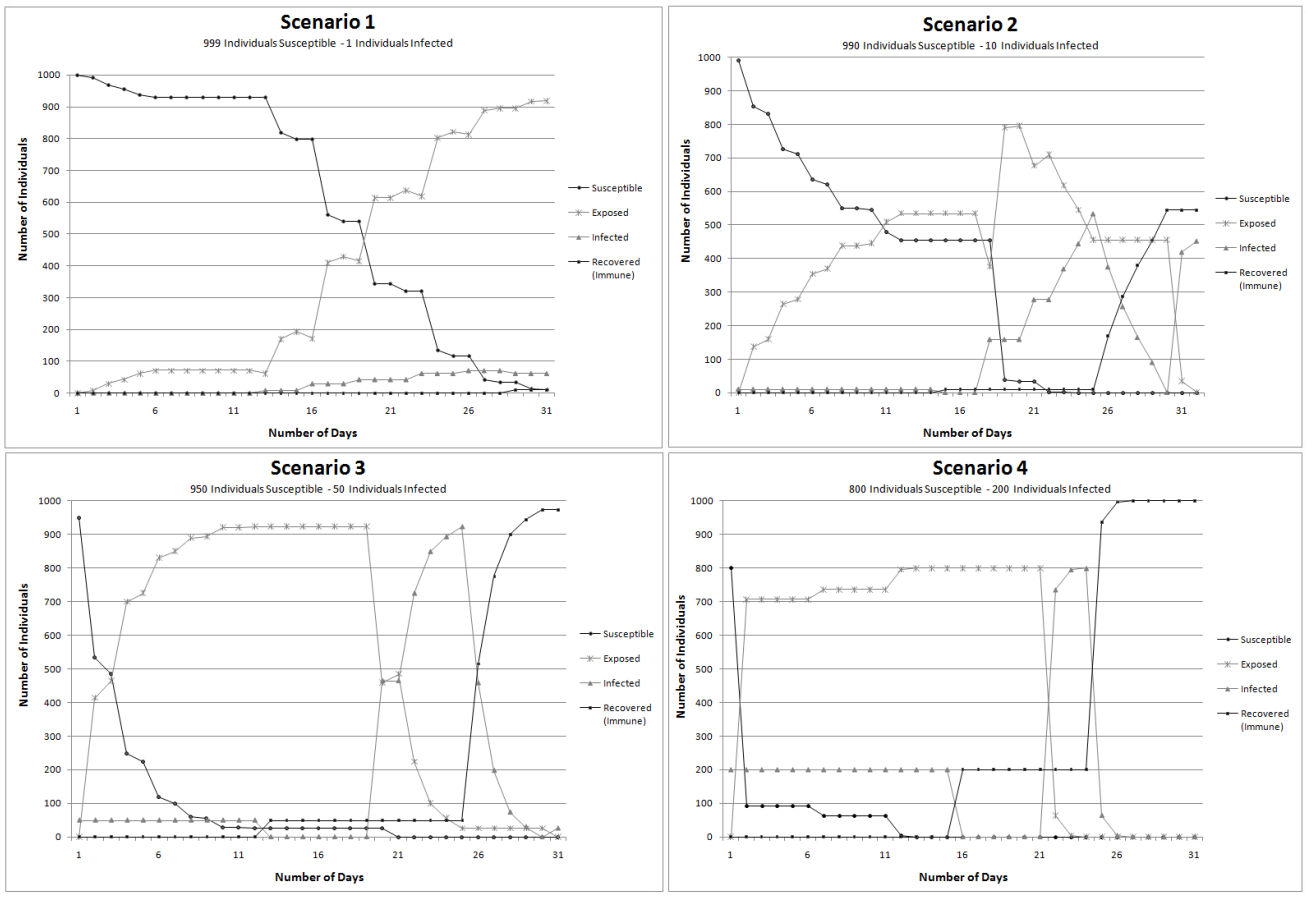
**Graphical representation of the variation in number of cases of Susceptible-Infected-Exposed-Recovered individuals within the population and for the simulation outcomes for four scenarios**.

These other scenarios were performed in order to obtain more insights about the dynamics of the disease spread under diverse circumstances. For this purpose the amount of initial ratio between susceptible and infected individuals in a specific population was modified for each scenario; these scenarios also allowed the model performance evaluation. Figure 10 demonstrates different rates progress in the spread of the disease, due to changes in the initial number of infected individuals.

Comparing Scenario 1 and Scenario 2 it is evident that the exponential increase in the population exposed is almost similar, but the time frame changes significantly. This suggests that in a close population interacting on daily basis, the probability of getting the infection increases proportionally to the number of the infectious agents in the environment. For the Scenario 3 and Scenario 4 the amount of initial infected individuals has a considerable influence in the spreading process of the epidemic. Although Scenarios 3 and 4 have respectively 5.3% and 25% of the initial population infected, the spreading phenomenon is not significantly different. In Scenario 3 after the third day, 70% of the entire population of susceptible individuals had been exposed to the disease and in Scenario 4 this spreading process occurred between the first two days.

Assessing the results under the different scenarios, *Scenario 1 *provided results closely similar to those observed in real events of measles epidemic; as a reference for this affirmation, the 107 cases of measles reported in 1997 in some parts of Metro Vancouver area [38]. To compare total infected individuals in Scenario 1 against the number of infected individuals reported in 1997, the time frame simulation used was sixty days. The results demonstrated that with one infected person at the beginning of the outbreak, 90% of the entire population, used in the simulation, is exposed to the disease after a time lapse of thirty days. In reality 107 cases were reported after sixty days and Scenario 1 reported 139 infected people after forty five days; the difference in the outcomes from Scenario 1 and the numbers described in reality is due to a series of reasons. First, the geographic area of individuals' interaction is a limited (Figure 4) and second, the population of individuals is closed. Finally, the simulation implemented in this study does not take into account any precaution measure to immunized susceptible individuals in order to avoid the disease and decrease the number of infected individuals.

The other reasons why the simulation outcome of the measles spread is slightly different from the one that occurred in 1997, is the fact that population was not divided by age groups, and also that the entire population taken in the model was considered as susceptible. In reality this is not the case due to a high percent of population has been vaccinated for this kind of diseases. The age factor is a very important parameter to consider due to young population possesses a higher probability to contract the disease and if taken into account this would alter the model outcomes. Furthermore, this study did not have access to real data on the individual measles cases due to confidentiality issues. In order to improve the accuracy of this model in further developments, it is important to divide the population into different age groups, and provide the information on vaccination status of each individual. In terms of improving agent behaviour, addition of the capability for an agent to have a degree of choice or willingness to stay at home and stop moving at the appearance of the first symptoms, would be beneficial. Such individuals help to slow down the disease spreading process and the epidemic would be modeled more realistically.

### Sensitivity Analysis of Model Outcomes

In order to assess the impact of the parameters and decision rules within the model, a sensitivity analysis (SA) was performed to determine how model is sensitive to changes in the parameters value. Sensitivity analyses are necessary to explore the behaviour of complex system models, because the structural complexity of the modeled process and the model is coupled with a high degree of uncertainty in estimating the values of many of the input parameters [43]. A sensitivity analysis quantifies how changes in the values of the input parameters alter the value of the outcome. There are two classes of SA techniques – univariate and multivariate [43]. In the univariate SA the model outcome is analyzed with respect to the variation of one parameter at a time whereas the other parameters of the system remain constant. The multivariate SA is concerned with systematically varying multiple input parameters and determining the impacts on the analyzed outcome [44,45].

For this study purposes a univariate technique was used. The first parameter of the sensitivity analysis is the *rate of infection *based on population density, and it was performed using four different scenarios: A, B, C, and D to initialize the simulation. Table 1 provide the variations of the *rate of infection *parameter based on population density low/medium/high. The second parameter for sensitivity analysis was related to the variations to *time spent for different activities*, was performed using three different scenarios: E, F, and G. The variations of overall *time *that individuals *spent for different activities *(e.g. working and/or studying, going to shopping malls, etc), are presented in Table 2. The disease spread simulations for the SA were produced for temporal intervals of 30-days using a one-hour time step. A closed population of 1000 individuals was chosen, where 999 individuals were susceptible and 1 individual was infected. The model outputs were then visually compared for all these scenarios to see how parameters impact the model performance.

**Table 1:** Set of values used to evaluate sensitivity to changes in rate of infection based on population density

**Scenario**	**Population Density**	Rate of Infection (%)*
A	Low	30
	Medium	50
	High	90

B	Low	90
	Medium	50
	High	30

C	Low	50
	Medium	30
	High	90

D	Low	40
	Medium	60
	High	70

* Percentage of EXPOSED individuals from the total SUSCEPTIBLE ones within the perimeter of contagion (Pc)

**Table 2:** Set of values used to evaluate sensitivity to changes in time spent for different activities

**Scenario**	**Activity**	Time Spent (Hours)*
E	Commuting	2
	Work	4
	Leisure	4

F	Commuting	2
	Work	2
	Leisure	4

G	Commuting	2
	Work	4
	Leisure	2

* Time spent by 30% of the individuals while the other 70% will stay for eight hour working/studying in the same location.

**Figure 11:**
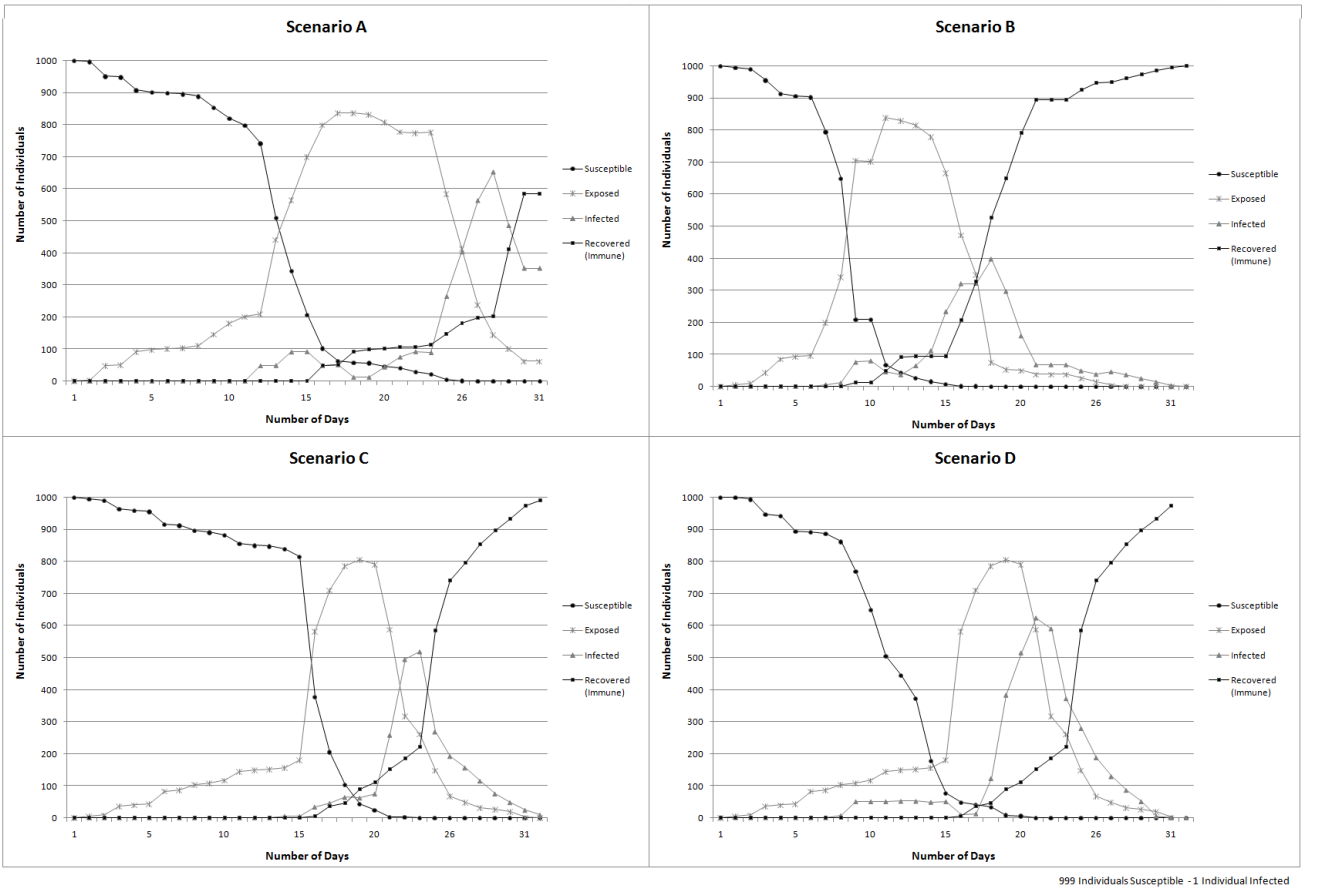
**Graphical representation of model sensitivity to changes in rate of infection based on the population density**. Simulation outcomes for scenarios A, B, C, and D.

**Figure 12:**
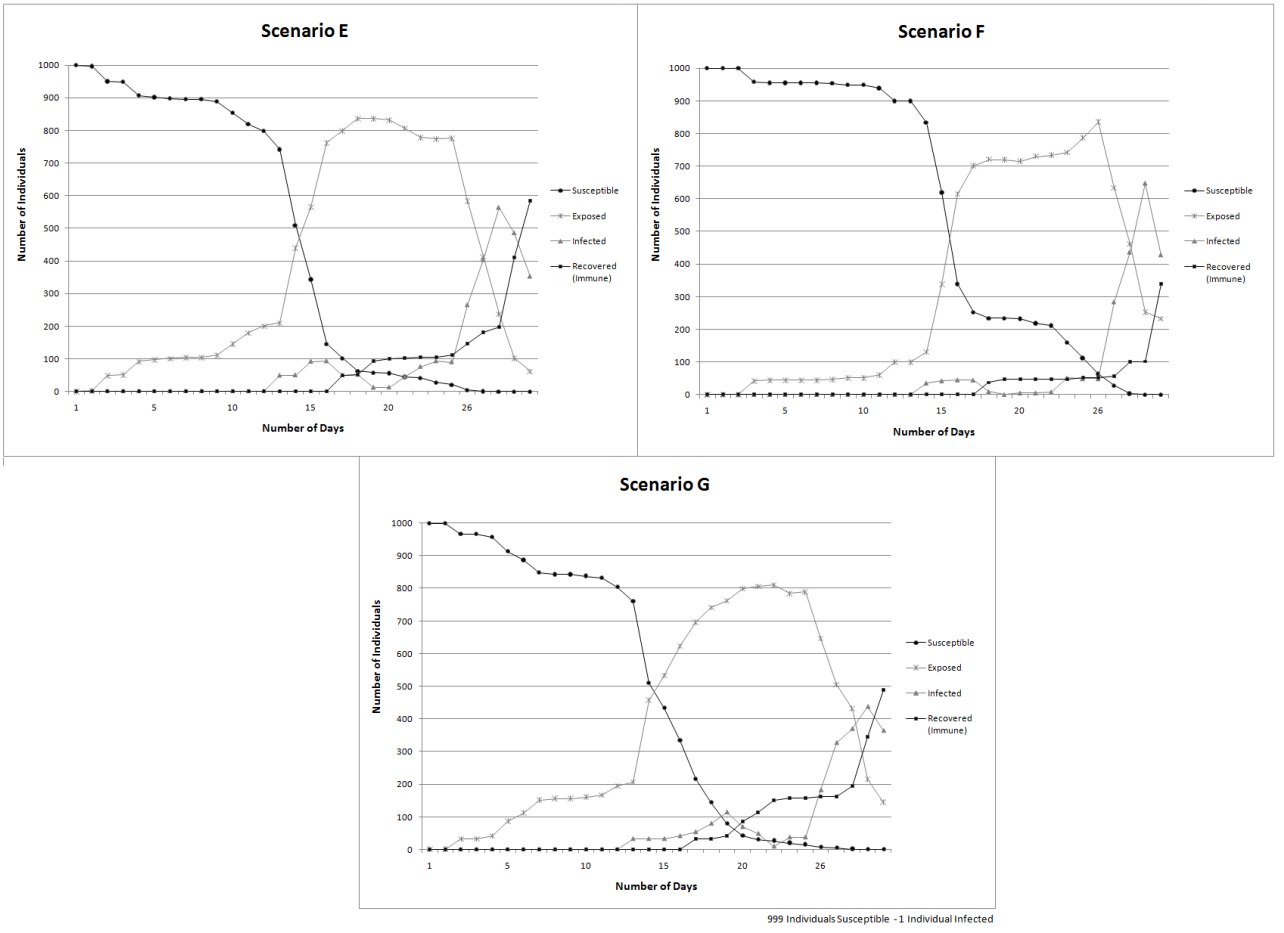
**Graphical representation of model sensitivity to changes in time spent for different activities**. Simulation outcomes for scenarios E, F, and G.

The simulation results obtained are used to evaluate the sensitivity of the model output to variations in the *rate infection *and they are presented in Figure 11, and sensitivity to changes in *time spent for different activities *are provided in Figure 12.

Figure 11 presents the number of daily exposures, infections and recoveries for a measles epidemic over a period of thirty days for the four scenarios – A, B, C and D. These results indicated that the model is sensitive to the *rate of infection *parameter, based on the population density. Comparing Scenario A and B it can be observed that changing the rate of infection from 30% to 90% in areas with low population density increased the number of individuals exposed to the disease in a shorter period of time. Evaluating Scenario A and C where the rates of infection were changed from 50% to 30% in areas with medium population density, it can be observed that the number of individuals exposed to the disease did not increase at the same rate. In Scenario A the greatest increase in the number of exposed individuals was observed in the day sixteen, meanwhile for Scenario C it occurs during the day twenty. Comparing Scenarios A and D smaller changes of the rate of infection based on population density lead to different simulation outcomes. Even though, the number of individuals exposed seems similar in both scenarios until day fifteen, afterwards the disease progress changed significantly.

Figure 12 presents the number of daily exposures, infections and recoveries for a measles epidemic over a period of thirty days for the three scenarios – E, F, and G. The results indicate that the model outcomes vary when the time spent by individuals in different daily activities changes. In Scenario E and G, when 30% of the individuals in the simulation are set to spend four hours working/studying the number of individuals exposed to the disease appears to be similar although these individuals spent different amounts of time in leisure activities. However, when the time spent working/studying is changed to two hours as in Scenario F, the disease progression varies by slowing down the process of spread among individuals trough the time.

## Conclusion

This study proposes a GIS-agent based model that simulates the outbreak of a communicable disease, in an urban area where different activities take place during a daily citizens' routine.

The results of disease propagation simulation indicate that the model is successfully able to generate various scenarios of an outbreak in complex and realistic geographic urban settings by incorporating movement in the agent entities. The addition of mobility allow realistic emulation of daily behaviours of individuals of a population that interact among themselves and that perform stationary activities in fixed spatially located areas after moving from one place to another. The model implemented in this study can be extended to incorporate parameters such as population gender, age, and ethnicity in order to introduce levels of susceptibility in different groups of individuals. Likewise, decisions taken by infected individuals such as stay at home to avoid the contact and spread of the disease can be included. The advantage of the GIS-AB model designed in this study is that any other communicable disease spread can be simulated by simple adjusting the modeled disease timeline and/or the infection model and modifying the transmission process.

The dynamics of the spread were implemented for the case of measles propagation and analyzed using the simulation outcomes. The output results from the behaviour of the disease spread demonstrated that global mixing in a closed population produces that almost the total number of individuals in the simulation becomes exposed after twenty five days. Furthermore, this study examined the impact of model parameters on the generated model outputs by determining the level of sensitivity to changes in the *rate of infection *based on population density and changes in *time spent for different activities *throughout seven different scenarios. Theses analyses are important since uncertainties embedded in the model outcomes are often either ignored or not adequately addressed. The proposed modeling approach offers a mean to analyze "what if" scenarios in case of a disease spread at a city-scale.

Some limitations of this model are with respect to model validation. The lack of information and real geographical location of the individual cases occurred during the 1997 outbreak and how these were treated is making this model theoretical. Often this type of data is not available due to confidentiality reasons, making very difficult to perform the model validation. Computational limitations are also an issue because not all the population can be considered given the limited computer memory capacity. This consequently affects the number of contacts between individuals within the city that can be simulated; increasing the interaction to such a point that the infection progress can appear faster than it may happens in reality, as individuals interact only through a limited network of transportation. Further work on model improvement includes agents endowed with additional attributes that allow a better insight of different groups of populations (e.g., ages, gender, ethnic group, etc), their daily habits and interactions among them, as well as degrees of age group susceptibility.

The model presented is a prototype that can be used as a laboratory to test possible outcomes and scenarios under a contagious disease outbreak at city-scale and with variations of different model parameters settings. The outcomes of the model simulations allow stating the importance of achieving sufficient knowledge about the spatial interactions of individuals and their contact networks. Further improvements to this approach would help to model and analyze the risk of disease spread through socially connected groups. Likewise, the results of this simulation can help to improve comprehension of the disease spread dynamics and to take better steps towards the prevention and control of an epidemic.

## Competing interests

The authors declare that they have no competing interests.

## Authors' contributions

The second author provided the initial ideas and model conceptualization. The first author programmed and coded the software routines and developed the GUI that permitted the model implementation. Both authors were involved equally in the design, development and implementation of the model as well as in the writing of the paper.

## References

[B1] Connolly M, Gayer M, Ryan M, Salama P, Spiegel P, Heymann D (2004). Communicable diseases in complex emergencies: impact and challenges. Lancet.

[B2] Fuks H, Duchesne R, Lawniczak AT (2005). Spatial correlations in SIR epidemic models. WSEAS MATH 2005; Cancun, Mexico.

[B3] Outbreak of Measles Epidemic in UK. http://www.medindia.net/news/view_news_main.asp?x=11342.

[B4] Mumps Outbreak Continues. http://www.gov.ns.ca/news/details.asp?id=20070413002.

[B5] Mumps: Coming Soon to A Place Near You. http://www.fraserhealth.ca/HealthTopics/CommunicableDiseases/mumps.

[B6] Ching Fu S, Milne G (2003). Epidemic Modelling Using Cellular Automata. ACAL2003: The First Australian Conference on Artificial Life; Canberra, Australia.

[B7] Kleczkowski A, Grenfell BT (1999). Mean-field-type equations for spread of epidemics: The 'small world' model. Physica A.

[B8] Sattenspiel L (2003). Infectious diseases in the historical archives: a modeling approach. Human Biologists in the Archives: Demography, Health, Nutrition and Genetics in Historical Populations.

[B9] Bauch CT, Brauer F, Driessche Pvd, Wu J (2008). The Role of Mathematical Models in Explaining Recurrent Outbreaks of Infectious Childhood Diseases. Mathematical Epidemiology.

[B10] Bian L, Liebner D, Maguire D, Goodchild MF, Batty M (2005). Simulating spatially explicit networks for dispersion of infectious diseases. GIS, Spatial Analysis and Modeling.

[B11] Kermack W, McKendrick A (1927). A Contribution to the Mathematical Theory of Epidemics. Proceedings of the Royal Society of London A.

[B12] Di Stefano B, Fuks H, Lawniczak AT (2000). Object-Oriented Implementation of CA-LGCA Modelling Applied to the Spread of Epidemics. 2000 Canadian Conference on Electrical and Computer Engineering, IEEE.

[B13] Sirakoulis GC, Karafyllidis I, Thanailakis A (2000). A cellular automaton model for the effects of population movement and vaccination on epidemic propagation. Ecological Modelling.

[B14] Zhen J, Quan-Xing L (2006). A cellular automata model of epidemics of a heterogeneous susceptibility. Chinese Physics.

[B15] Bagni R, Berchi R, Cariello P (2002). A comparison of simulation models applied to epidemics. Journal of Artificial Societies and Social Simulation.

[B16] Patlolla P, Gunupudi V, Mikler AR, Jacob RT (2004). Agent-Based Simulation Tools in Computational Epidemiology. 4th International Workshop, International Conference on Innovative Internet Community Systems (I2CS '04); June 21–23; Guadalajara, Mexico.

[B17] Gordon TJ (2003). A simple agent model of an epidemic. Technological Forecasting and Social Change.

[B18] Dunham JB (2005). An Agent-Based Spatially Explicit Epidemiological Model in MASON. Journal of Artificial Societies and Social Simulation.

[B19] Epstein JM, Axtell RL (1996). Growing Artificial Societies: Social Science From the Bottom Up.

[B20] Gilbert N, Troitzsch K (2005). Simulation for the Social Scientists.

[B21] Epstein J, Cummings D, Chakravarty S, Singa R, Burke D (2004). Toward a Containment Strategy for Smallpox Bioterror: An Individual-Based Computational Approach.

[B22] Chen LC, Kaminsky B, Tummino T, Carley KM, Casman E, Fridsma D, Yahja A (2004). Aligning Simulation Models of Smallpox Outbreaks.

[B23] Eidelson BM, Lustick I (2004). VIR-POX: An Agent-Based Analysis of Smallpox Preparedness and Response Policy. Journal of Artificial Societies and Social Simulation.

[B24] Carley KM, Fridsma DB, Casman E, Yahja A, Altman N, Chen L-C, Kaminsky B, Nave D (2006). BioWar: Scalable Agent-Based Model of Bioattacks. IEEE Transactions on Systems, Man, and Cybernetics, Part A: Systems and Humans.

[B25] Bomblies A, Duchemin J-B, Eltahir E (2008). Hydrology of malaria: Model development and application to a Sahelian village. Water Resources Research.

[B26] Keeling M (1999). The effects of local spatial structure on epidemiological invasions. Proceedings of the Royal Society of London B.

[B27] Chowell G, Hyman J, Eubank S, Castillo-Chavez C (2003). Scaling laws for the movement of people between locations in a large city. Physical Review E.

[B28] Bian L (2004). A conceptual framework for an individual-based spatially explicit epidemiological model. Environment and Planning B: Planning and Design.

[B29] Yang Y, Atkinson PM (2005). An Integrated ABM and GIS Model of Infectious Disease Transmission. Computers in Urban Planning and Urban Management – CUPUM'05; 29 June – 1 July; London, England.

[B30] Ferguson NM, Keeling MJ, Edmunds WJ, Gani R, Grenfell BT, Anderson RM, Leach S (2003). Planning for smallpox outbreaks. Nature.

[B31] Bian L, Liebner D (2007). A network model for dispersion of communicable diseases. Transactions in GIS.

[B32] Simoes JM (2005). Modelling the Spreading of Infectious Diseases using Mobility Networks. CUPUM 2005 The Ninth International Conference on Computers in Urban Planning and Urban Management; July; London, UK.

[B33] EpiSIM – Software for Spatial Epidemic Simulation. http://www.casa.ucl.ac.uk/joanamargarida/websiteProject/.

[B34] Bouden M, Moulin B, Gosselin P (2008). The geosimulation of West Nile virus propagation: a multi-agent and climate sensitive tool for risk management in public health. International Journal of Health Geographics.

[B35] Li Z, Hayse V, Hlohowskyj I, Smith K, Smith R (2005). Agent-based Model for Simulation of West Nile Virus Transmission. Proceedings of the Agent 2005 Conference on Social Dynamics: Interaction, Reflexivity and Emergence; June 26–28; Chicago, USA.

[B36] Vaccine-Preventable Diseases Measles. http://www.phac-aspc.gc.ca/im/vpd-mev/measles-eng.php.

[B37] Yang Z, Haas Pd, Wachmann C, Soolingen Dv, Embden Jv, Andersen A (1995). Molecular epidemiology of tuberculosis in Denmark in 1992. Journal of Clinical Microbiology.

[B38] Bell A, King A, Pielak K (1997). Epidemiology of measles outbreak in British Columbia- February 1997. Canada Communicable Disease Report.

[B39] Census Canada 2001. http://www.statcan.gc.ca/.

[B40] Metro Vancouver Regional District. http://www.metrovancouver.org.

[B41] The Greater Vancouver transit authority. http://www.translink.bc.ca.

[B42] Repast Simphony. http://repast.sourceforge.net/index.html.

[B43] Kocabas V, Dragicevica S (2006). Assessing cellular automata model behaviour using a sensitivity analysis approach. Computers, Environment and Urban Systems.

[B44] Iman RL, Helton JC (1988). An Investigation of Uncertainty and Sensitivity Analysis Techniques for Computer Models. Risk Analysis.

[B45] Crosetto M, Tarantola S, Saltelli A (2000). Sensitivity and uncertainty analysis in spatial modelling based on GIS. Agriculture, Ecosystems and Environment.

